# Breast tumor segmentation using neural cellular automata and shape guided segmentation in mammography images

**DOI:** 10.1371/journal.pone.0309421

**Published:** 2024-10-01

**Authors:** Mudassar Ali, Tong Wu, Haoji Hu, Tariq Mahmood

**Affiliations:** 1 College of Information Science and Electronic Engineering, Zhejiang University, Hangzhou, China; 2 University of Illinois Urbana-Champaign Institute, Zhejiang University, Hangzhou, Zhejiang, China; 3 Artificial Intelligence & Data Analytics Lab, CCIS Prince Sultan University, Riyadh, Kingdom of Saudi Arabia; 4 Faculty of Information Sciences, University of Education, Vehari Campus, Vehari, Pakistan; Shijiazhuang Tiedao University, CHINA

## Abstract

**Purpose:**

Using computer-aided design (CAD) systems, this research endeavors to enhance breast cancer segmentation by addressing data insufficiency and data complexity during model training. As perceived by computer vision models, the inherent symmetry and complexity of mammography images make segmentation difficult. The objective is to optimize the precision and effectiveness of medical imaging.

**Methods:**

The study introduces a hybrid strategy combining shape-guided segmentation (SGS) and M3D-neural cellular automata (M3D-NCA), resulting in improved computational efficiency and performance. The implementation of Shape-guided segmentation (SGS) during the initialization phase, coupled with the elimination of convolutional layers, enables the model to effectively reduce computation time. The research proposes a novel loss function that combines segmentation losses from both components for effective training.

**Results:**

The robust technique provided aims to improve the accuracy and consistency of breast tumor segmentation, leading to significant improvements in medical imaging and breast cancer detection and treatment.

**Conclusion:**

This study enhances breast cancer segmentation in medical imaging using CAD systems. Combining shape-guided segmentation (SGS) and M3D-neural cellular automata (M3D-NCA) is a hybrid approach that improves performance and computational efficiency by dealing with complex data and not having enough training data. The approach also reduces computing time and improves training efficiency. The study aims to improve breast cancer detection and treatment methods in medical imaging technology.

## 1 Introduction

Globally, people widely recognize cancer as a leading cause of death. Worldwide, breast cancer is the most prevalent type of cancer that affects women [1]. Lesions are important in the setting of breast cancer because they serve as critical indicators for detecting and comprehending the illness. These lesions show aberrant tissue alterations in various patterns, which help to differentiate various forms of breast cancer [2].

IDC is the most common form of breast cancer: invasive ductal carcinoma. As such, this type highlights the invasion of malignant cells into the surrounding tissues of the breasts. ILC stands for invasive lobular carcinoma, which means that the tumor can spread from milk-producing ducts to nearby tissues. Ductal Carcinoma in Situ, on the other hand, is an early-stage duct cancer that is defined by abnormal cells that have not yet spread to the epithelium of a breast duct [3].

Mammography is a very essential imaging modality for the early diagnosis of breast cancer. It uses low-dose X-rays to detect irregularities in breast tissue. The tool mentioned is one of the most applied ways through which breast screening takes place and has a high degree of effectiveness in detecting diseases at an early stage of breast cancers, even before symptoms can manifest themselves [4, 5]. Improved early detection rates and diagnostic accuracy could be regarded as some of the key targets in mammography and other imaging strategies, such as MRI developments [4].

Integration of Deep Learning algorithms [6] and Machine Learning [7] has been responsible for recent developments in the area of breast cancer confirmation. These machine learning algorithms use complex procedures to process bulk medical data in search of complicated trends and variance, hence increasing the accuracy of detection [8]. Deep Learning, which is a subset of Machine Learning, uses neural networks for image recognition and is astoundingly brilliant at processing mammograms, ultrasounds, and MRIs. [9]. Nonetheless, most seasoned deep learning models require substantial computational power, which limits their use in low-resource settings like developing countries and rural medical centers [10–12]. Moreover, although MRI provides very detailed imaging, it is very expensive, time-consuming, and therefore prone to false positives that may lead to unnecessary biopsies [13].

Neural Cellular Automata (NCA) [14] offer an alternative method for segmenting medical images. NCAs, in contrast to deep learning models, function by relying on local cell interactions, which makes them both efficient and adaptable. However, classical NCA models lack the processing power to deal with high-resolution images. Segmentation tasks have shown that integrating shape priors may be beneficial. Recently, it has been illustrated that the incorporation of shape information improves the accuracy of segmentation by delineating boundaries more precisely [15, 16]. Chen et al. [17] integrated a segmentation model with shape constraints by utilizing a deep Boltzmann machine. Superpixels have captured the shape information well enough to make correct boundary predictions for many segmentation tasks [18, 19]. The developed model aims to improve the performance of NCAs for the medical image segmentation process, thereby assisting the whole process in attaining high accuracy and effectiveness in breast cancer diagnosis. It couples the framework with shape-guided segmentation to enhance model efficiency and effectiveness. As a result, the methodology is furthering the challenges experienced in low-resourced regions by highlighting the need for sophisticated computational tools that aid in diagnosis.

### 1.1 Objectives of the proposed method

**1. Combining NCA and SGS:** To integrate the techniques of NCA and SGS so that this integrated technique brings better accuracy in segmentation, which remains consistent across different radiology practices or sub-populations of patients.

**2. Enhancing Efficiency and Ensuring Quality Control:** Efficiency and quality control: The level of care and thoroughness in an automated quality control process should be maintained while maximizing computational efficiency to prevent the emergence of extra expenses.

**3. Providing Radiologists with State-of-the-Art Instruments:** Leverage the power of consequently highly advanced computational tools to enable radiologists with better breast cancer diagnostics.

**4. Dealing with constraints on available resources:** Create a universally accessible solution that can be adjusted to different healthcare settings, thereby helping to decrease healthcare inequalities.

**Fig 1:**
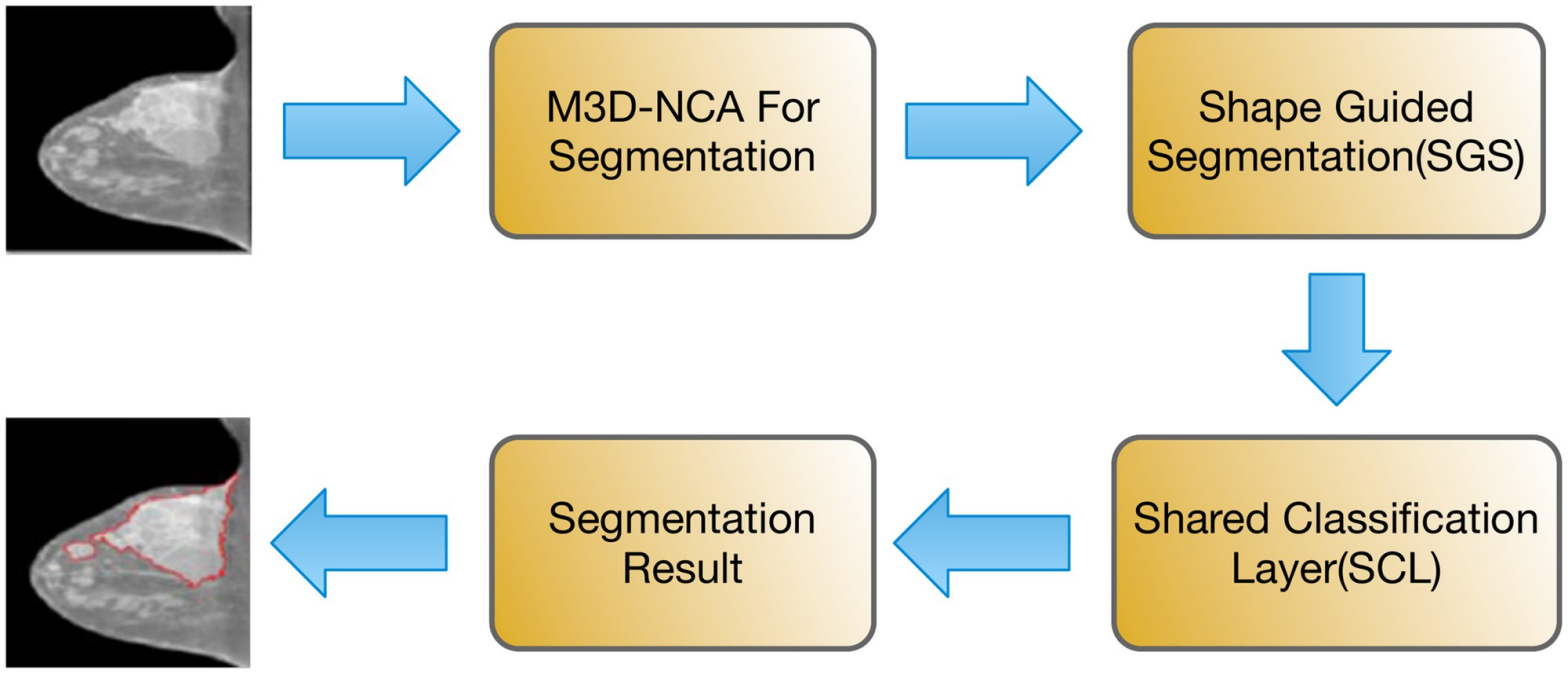
Flowchart of functional units.

Fig 1 depicts the block diagram of the proposed approach, which introduces multiple innovative techniques for segmenting breast tumors. The proposed model guarantees consistent accuracy in segmenting regardless of changes in medical practices or patient populations, strikes a balance between model performance and computational efficiency, improves radiologists’ diagnostic abilities, and adjusts to different healthcare settings.

The organization of this document is delineated as follows: The study’s important earlier research is discussed in Section 2. Section 3 explores the techniques used to identify and separate breast abnormalities. The empirical dataset and the results are displayed in Section 4. The results, future research directions, and limitations of the study are outlined in Section 5. The concluding remarks of this paper are presented in Section 6.

## 2 Related work

The subsequent segments encompass focused research on the segmentation of breast cancer in mammography and general image analysis techniques that are closely associated with the proposed field of study.

### 2.1 A general method for segmentation in medical imaging

Medical imaging has advanced greatly with deep learning technologies, specifically deep convolutional neural networks (CNNs), making segmentation a key area of computer vision research [20]. Thresholding, clustering, edge detection, and the Lattice Boltzmann method have improved traditional segmentation methods. These methods define and classify image regions for accurate image analysis and tumor diagnosis [21].

Deep learning has been popular in this field because it can autonomously learn and identify visual features, outperforming manual segmentation [22]. The U-net architecture excels at semantic segmentation in medical imaging, improving diagnostic accuracy through precise image feature evaluation [23].

In the particular domain of mammography segmentation, several techniques have demonstrated notable efficacy:

#### 2.1.1 Certain effective mammogram segmentation methods

**A. Edge Detection:**

The conventional methodology employs gradient matrices to generate a binary mask that demarcates the boundaries of an image [24], emphasizing notable variations in pixel intensity. Identifying item borders inside the image is essential [25].

**B. Thresholding:**

An extensively employed technique in medical imaging, this method separates the foreground from the background by utilizing Otsu’s adaptive thresholding [26], histogram-based, and global fixed thresholding [27, 28]. These techniques effectively identify problematic regions in mammograms through the analysis of fluctuations in pixel intensity [29].

**C. Watershed Transformation:**

This technique utilizes the metaphor of a landscape with peaks and valleys to delineate the boundaries of different regions [30]. It employs a process similar to flooding terrain to efficiently divide the image into its components [31].

**D. Clustering:**

In contrast to region-growing techniques [32], clustering groups pixels according to similarity metrics as opposed to predetermined seed points [33]. Prominent examples include imprecise C-means and K-means, the latter of which permits a more gentle classification and is more adaptable to the subtleties of mammogram images [34].

**E. Utilizing Atlas-based Segmentation:**

The segmentation of new images is guided by a database of pre-segmented images in this sophisticated method [35, 36]. Image registration is utilized to align and modify these templates to the particular anatomy depicted in the mammogram [37].

As a whole, these methodologies highlight the rapid progression of medical image segmentation, placing particular emphasis on the transition to automated, deep learning-driven strategies that offer enhanced accuracy and dependability in the field of medical diagnostics.

### 2.2 Breast tumor segmentation based on classical method

Certain researchers have devised techniques to detect breast cancer lesions at an early stage. Utilizing the developed methodologies, masses in breast cancer images have been segmented. The approach comprises the following methods: marker-controlled watershed, threshold, active contour model, region-growing, watershed, template-matching, and level set [38, 39]. The classical segmentation method is comprised of three groups: threshold-based segmentation, edge-based segmentation, and region-based segmentation. It is based on the pixel values of an image [40]. Some studies are explained below that use threshold-based segmentation or edge-based segmentation or region-based segmentation.

The comparative analysis of segmentation algorithms for the detection of microcalcifications in mammography images was undertaken by Podgornova et al [41]. In the current study, benchmarking of three segmentation techniques has been done: watershed, mean shift, and k-means. The dataset used in the experiment consisted of 250 mammography images obtained from the Mammographic Image Analysis Society database. The results obtained from the watershed segmentation method were an accuracy rate of 18.0%, and in the detection results, 94.0% were false. The mean shift method detected the true detection rate at 39.22% and the false detection rate at 60.8%. Meanwhile, k-means segmentation showed a true detection rate of 42.8% and a false detection rate of 57.2%.

B.N. Beena et al [42] proposed a technique for segmenting mammogram images by waters idealist segmentation and classifying cancer present using the k-NN. The standard classification k-NN technique was used; the method followed to do it was the extraction of GLCMs through the Halarick texture characteristics from an available dataset of 60 mammogram images in the MIAS dataset; a Sober filter was used for noise reduction, which finally gave a segmentation of 83.33% and accuracy of 83.33.

Singh et al [43] presented the segmentation technique for breast cancer, making use of global thresholding and region merging. Wiener filtering technique was used to reduce the Gaussian noise in the present study, and in order to normalize the image, a histogram shrinkage technique was employed. Otsu’s approach for global thresholding was underpinned in the proposed to give an accuracy at 82.0% with a corresponding error rate of 18.0%, applied to a dataset of 50 images of mammography.

The research proposed by Syeda et al [44] was on automatic pectoral muscle segmentation in mammography images. This study isolates and removes the pectoral muscles in the mammograms of a breast by using global threshold and weak boundary detection techniques. A convex-hull segmentation is then performed. This technique results in an accuracy of 92.86% in segmentation from the MIAS database with a median filter for the removal of noise. Also, 4.97% of the photos received good segmentation.

In addition, Andrik et al [45] and his research group presented a methodology for fully automatic breast boundary and pectoral muscle segmentation in mammography. In the present study, the contour boundaries of the pectoral muscle were identified using Canny edge detection. In order to remove the noise, median and anisotropic diffusion filters were also performed. The method as described above returned Dice similarity coefficients within the range of 97.8% to 99.2% with the use of datasets based on the MIAS, INbreast, and the Breast Cancer Digital Repository databases.

Another comprehensive framework for the enhancement, mass segmentation, and classification of mammography images was proposed by Al-Najdawi et al. [46]in which the methodology that the researchers used incorporated a number of enhancement techniques such as Clare and median filters, among others, in order to improve the performance of breast ROI detection. On a dataset of 1300 mammogram images acquired from King Hussein Cancer Center and Jordan Hospital, it achieved sensitivities and specificity rates of 96.2% and 94.4%, respectively. On a separate note, the classifier achieved accuracies for mammogram mass calcification of between 81.4% and 94.1%, with a segmentation accuracy of 90.7%.

### 2.3 Breast tumor segmentation based on deep learning approaches

The process of segmenting breast cancers was accomplished by using deep learning methodologies implemented on diverse neural networks. ome models, on the other hand, use deep learning techniques for breast tumor segmentation, such as U-Net [47], SegResNetVAE [48], DynUNet [3], UNETR [49], RF-Net [50], and MDA-Net [51]. Such models have shown impressive effectiveness with regard to their ability to accurately obtain segmentation results.

The architecture of U-Net incorporates an encoder to acquire comprehensive contextual representations and a decoder to make semantic predictions. Skip connections are used to retrieve incomplete spatial data. A dice score of 86.60% and an accuracy of 90.73% were attained [47]. A modified U-Net with residual connections, autoencoder regularization, and unique Encoder Block and Variational Autoencoder Branch outperforms the standard version. The Autoencoder Truncation branch boosts the dice score to 89.36% and accuracy to 92.78% as per published data. [48].

The DynUNet, a dynamic variant of the U-Net paradigm, outperforms both U-Net and SegResNetVAE models with 93% accuracy and 89.57% dice score [3]. UNETR, incorporating multi-head self-attention instead of 3D convolutions, achieves 90.73% accuracy and 89.59% dice score but falls short compared to DynUNet [49].

The RF-Net design uses an encoder-decoder architecture to enhance pixel predictions by feeding challenging pixel residual representations into encoder blocks. Achieving 92.92% accuracy and a dice score of 89.56%, this method is highly proficient in segmenting breast lesions [50].

MDA-Net features a multiscale fusion block with 1 × 1, 3x3, and 5x5 convolutions, addressing fixed receptive field issues and facilitating semantic feature extraction. MDA-Net enhances brain tumor segmentation by achieving 93.48% accuracy and a 90.25% dice score [51]. Deep learning methods, like ECNN with optimized loss functions, 3 × 3 filters, and BAT algorithm for binary segmentation, improve efficiency. A novel approach, sequential U-Nets, automates 3D brain tumor detection using a stacked U-Net design. ARU-GD method enhances computer learning with performance metrics [52]. Pan et al. [53] study introduces three new modules (PEM, SGA, and CSA) to enhance medical image segmentation in EG-TransUNet, a Transformer-based architecture.

Utilising MDU-Net, a Multi-Scale Densely Connected U-Net, to segment biomedical images. By incorporating novel multi-scale dense connections (MDC), their model successfully amalgamates adjacent feature maps, resulting in a 3.5% enhancement in segmentation performance. In liver tumor diagnosis from CT images, a novel approach combines U-Net with GW-CTO algorithm, outperforming alternatives by 4.3%-5.2%, with 85% learning efficiency [54, 55]. In eye tracking, Eye-UNet, incorporating an attention mechanism, excels in segmenting low-quality human eye images, outperforming other methods [56]. A compilation of conventional methodologies pertaining to the segmentation of breast tumors is presented in Table 1.

**Table 1:** Understanding the limitations of convention studies.

Ref.	Used Approaches	Performance Evaluation	Limitation
Mugahed et al. [57]	Gradient Boosting	Achieved 0.809 accuracy and 0.839 AUC for CKD progression prediction.	The study used a retrospective data set and may not be generalized to other populations or healthcare settings.
Vivek et al. [30]	cGAN	cGAN provides a high dice coefficient and IoU of 0.94 and 0.87, respectively.	The cGAN will successfully segment the full-shaped tumor but will misjudge the incomplete one if both are in the loose-fitting frame.
Olaf et al. [47]	U-Net	Achieved 0.837 accuracy and 0.806 dice score	Provides the valid DC score values (80.6%). Nevertheless, their values cannot match their pace compared to more intricate techniques.
Ali et al. [49]	UNETR	Achieved 0.929 accuracy and 0.895 dice score	UNETR’s transformer-based structure may require more training data and computational resources than conventional U-Net models due to its complexity.
Fabian et al. [3]	DynUNet	Achieved 0.930 accuracy and 0.896 dice score	DynUNet’s dynamic nature may increase computational intricacy compared to conventional U-Net models, as it adapts layers and filters based on input image size, potentially causing increased demands during training and inference.
Zhemin et al. [48]	SegResNetVAE	Achieved 0.927 accuracy and 0.893 dice score	SegResNetVAE requires more training data and computational resources due to the VAE component’s latent space representation and the complexity of the ResNet and VAE architectures.
Wang et al. [50]	RF-Net	Achieved 0.929 accuracy and 0.895 dice score	RF-Net’s performance may be hindered by high training data volume and increased processing demands due to its recurrent connections in medical images.
Ahmed et al. [51]	MDA-Net	achieved 0.934 accuracy and 0.902 dice score	MDA-Net’s performance can be affected by distribution differences between source and target domains, lack of overlap, hyperparameter selection, and adaptation complexity, affecting its ability to adjust and extrapolate efficiently.

## 3 Proposed methodology

**Fig 2:**
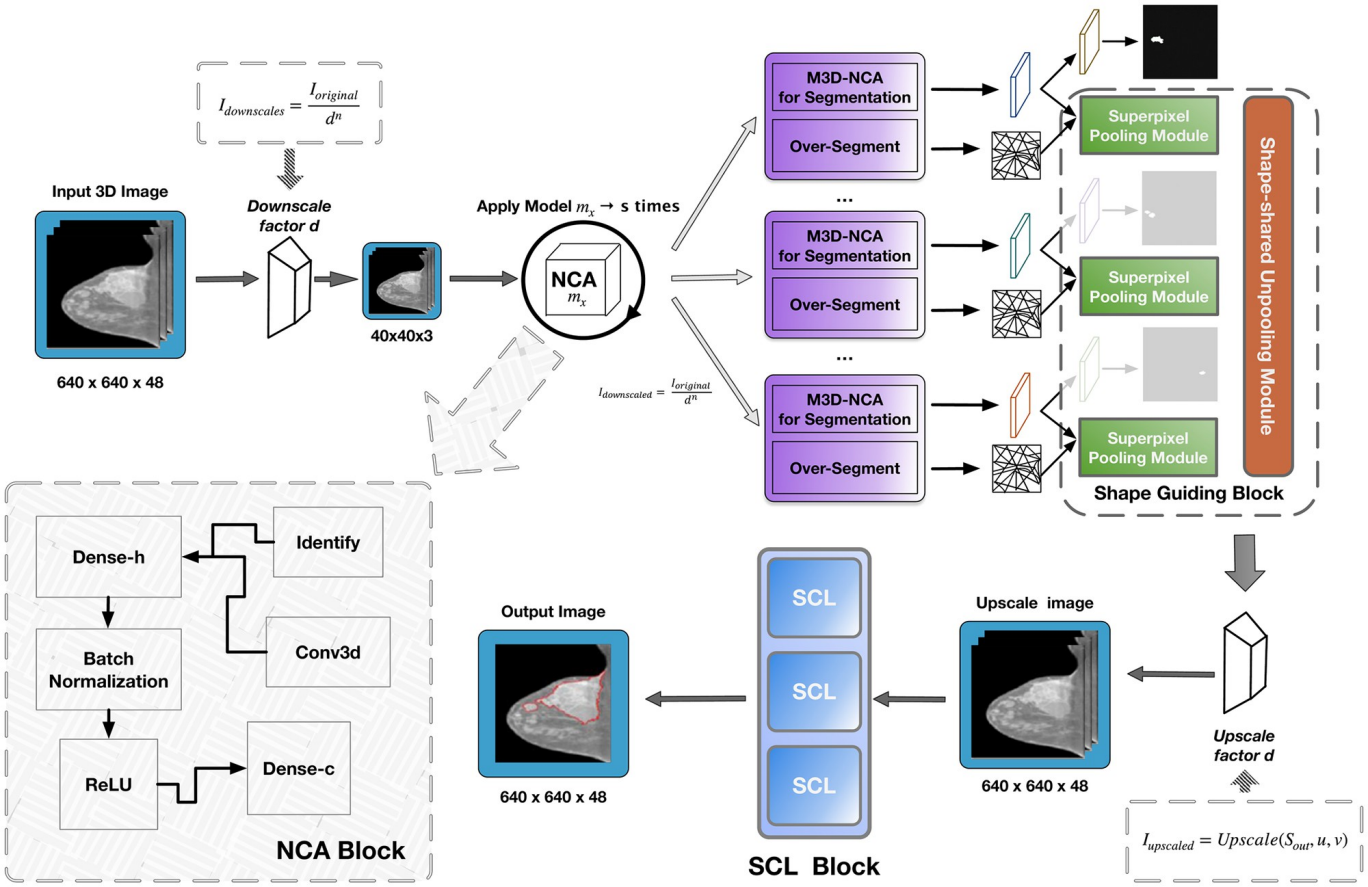
An all-encompassing model architecture that provides a graphical representation of the structure of the proposed network model.

The main aim of this paper is to improve the efficiency of Neural Cellular Automata in medical image segmentation in identifying occurrences of breast cancer. Addressing the issue of high V-RAM consumption at the training stage, Kalkhof et al [58] introduced downscaling factors and tunable layer numbers into the M3D-NCA pipeline. In the paper [32], Lin et al. revealed that SGS-based methods significantly improve the accuracy of segmentation, especially for locating blurred boundaries in medical images. The proposed model architecture of the research is more efficient due to the combination of the M3D-NCA framework with SGS. Moreover, effective use of components also enhanced its feature of improving the accuracy of segmentation and delineating tumor boundaries more precisely. Segmentation in medical images, more precisely in the area of breast cancer, might become difficult due to the fact that the shapes of tumors are complex and hugely variable, together with the accuracy required during the drawing of boundaries. Having this in mind, the following architectural design inherited some critical elements of the previous, as shown in Fig 2.

### 3.1 Rationale for using a 3D model for 2D data

This study is set to investigate the use of a three-dimensional model in analyzing two-dimensional image datasets for better feature extraction, boundary delineation, and accuracy on segmentation tasks. Clearly, the depth and contextual information in multiple planes are very important for accurate breast tumor segmentation. The approach will make use of 3D convolutions and 3D batch normalization to capture features that span along slices, which assist in improving the consistency of segmentation. It helps to stabilize the process of learning and decrease the effect of internal covariate shifts, improving the ability to generalize learned knowledge to new situations.

The 3D model brings the added functionality of handling multi-channel data, in which case correlation and interaction among different image channels help to derive accurate and reliable segmentation. The M3D-NCA framework efficiently utilizes the spatial context and manages data to produce high-quality segmentation results.

One of the advantages of using a 3D model is that it gives contextual information necessary for the accurate segmentation of structures in images. It can capture fine structures better, maintain consistent boundaries, and enhance learning ability. The ability of the 3D model to process volumetric data better enables it to learn and generalize from the data and hence perform better than models operating in 2D.

By integrating the sophisticated methods of SGS with 3D models, RUNet can include shape priors or other contextual information in a more comprehensive fashion. This can greatly boost the model’s ability to generate precise and dependable segmentation outcomes. On its own, the advantages brought by the 3D model are adequate for it to be utilized even when the input data is in the format of 2D slices.

### 3.2 M3D-neural cellular automata (M3D-NCA)

M3D-NCA represents a three-dimensional cellular automata model designed especially for medical imaging data. The software models the proliferation and interaction of cells in the three-dimensional grid by using cellular automata principles, and it is able to segment a breast tumor very efficiently. This process is started by the model through the generation of a grid of cells representing the three-dimensional volume of breast tissue. Following that, each cell updates its state according to the states of its neighbors based on the learned rules. The latter makes it possible for the model to iterate toward better segmentation of the tumor. The approach will ensure both an optimal usage of computational resources and high accuracy in tracing all fine details of breast tumor boundaries. The training process uses a combination of Dice Loss and Binary Cross-Entropy to drive better segmentation performance. Inherent adaptive learning of the evolution rules for cell states in M3D-NCA reinforces its strength toward robustness across variations in tumor appearance among different patients.

### 3.3 Shape-guided segmentation (SGS)

Shape-guided segmentation is one of the approaches that increases the accuracy of segmentation by making good use of tumor shape data available in advance. It is particularly efficient in medical imaging, especially when dealing with anatomic structures with consistent shapes. It uses advanced methods, such as SLIC over-segmentation techniques, to generate superpixels that may later be used as guides in order to further segment any given image. SGS enhances the accuracy of segmentations by shape information embedded within the segmentation model, ensuring that the result of the segmentations fits very tightly to the true anatomical boundaries. In this work, they introduce SGS into the M3D-NCA framework, which they refer to as the Shape-Guided Block (SGB). Then, the SGB consists of modules like Shape-Shared Unpooling, represented as SPM, and Superpixel Pooling as SUM. These modules correct deviations from the anticipated tumor shapes and thus improve the segmentation iteratively. This step-by-step integration will improve the accuracy of segmentation and enhance the model’s capability in handling indistinct or uncertain boundaries in medical images.

### 3.4 The proposed architecture of the model

This model architecture is configured to deliver an accurate breast tumor segmentation using sophisticated methodologies. Due to M3D-NCA and SGS, this method improves the accuracy and computational efficiency of segmentation. It exploits this advantage by using the rule-based evolution of cellular automata from M3D-NCA and uses it with accurate, shape-constrained guidance from SGS. This collaboration enhances the performance of the model in order to ensure its preciseness for the outlining of tumors and efficient computation within the bounds of the available resources. It has critical constituents in the architecture to assure dependable segmentation results. Algorithm-driven development in M3D-NCA with an accurate, shape-informed direction of SGS builds on the overall effectiveness to guarantee precise identification of tumor boundaries.

#### 3.4.1 Convolutional Base Block

The process of the experiment is such that it performs many different convolutional operations atop a Convolutional Base Block with a kernel size around *k*, embedded with a Dense Layer, 3D Batch Norm, and ReLU activation. This is a purposeful plan in a way that will be able to try out pooling the low-frequency data recursively to handle the V-RAM utilizations very efficiently. The operations can be expressed using the Eq 1.

$$\begin{array}{c} C_{\text{out}}=\text{ReLU}(\text{BatchNorm}(\text{Dense}(C_{\text{in}}\cdot K+b))) \end{array}$$ (1)

Where:

- *C*_in_ and *C*_out_ represent the input and output channels, respectively.
- *K* is the convolution kernel with a size of *k* × *k* × *k*.
- *b* is the bias term.
- *Dense*, *BatchNorm*, and *ReLU* represent the dense layer, 3D batch normalization, and ReLU activation function, respectively.

These connections are made by the dense layer; however, it is better to make a network where input or output nodes can be connected. It thus gives a network a chance to learn complex patterns. 3D Batch Normalization standardizes the inputs to reduce the changeability of the data distribution and make sure data is consistently distributed. The ReLU activation function introduces non-linearity into the network, thereby enabling the learning of complex patterns and resolution of issues such as the vanishing gradient problem. The Convolutional Base Block puts those elements together in a way that can improve model capacity, ensure consistent training, and add non-linearity for the acquisition of intricate patterns. This layer just effectively picks up the low-frequency data and optimizes the use of V-RAM, hence laying a base for the following layers and operations.

#### 3.4.2 Downscaling block and NCA Model Block

Downscaling block in large sizes of images is an important tool. It decreases the spatial dimensions of the input images and, therefore, reduces the computational load and memory required for the processing. In this way, it keeps model efficiency when dealing with larger datasets. It resizes the input images to the proper size before passing them to the next layers. This ensures that the greatest part of the structure and key features of the data is maintained at the same time as optimizing resource utilization. Mathematically, this downscaling process may be defined in Eq 2.

$$\begin{array}{c} I_{\text{downscaled}}=\frac{I_{\text{original}}}{d^{n}} \end{array}$$ (2)

where:

- *I*_original_ is the original dimension of the image.
- *d* is the downscaling factor.
- *n* is the number of downsampling iterations.

The following are some of the essential functions performed by the downscaling block in reducing the spatial information: The NCA (Neural Cellular Automata) Model Block plays a critical role in breast tumor segmentation. For the system to test and segment the images, it has been given some specific hyperparameters. The first layer has an increased kernel size, where *k* = 7, so that the receptive field can be wide. This helps to capture quite a good deal of contextual information. Then the following layers go on to have a smaller kernel size of *k* = 3, focusing on finer details and features.

The NCA Model Block convolves the input image through a sequence of convolutional layers, where the kernel size keeps reducing from the first to the last convolution layer in the block. In so doing, it attempts to embody both global and local feature variations to acquire a more reliable segmentation result. The formulaic definition of this block read Eq 3:

$$\begin{array}{c} O={\text{Conv}}_{k=3}\left(\text{ReLU}\left(\text{BatchNorm}\left({\text{Conv}}_{k=7}\left(I\right)\right)\right)\right) \end{array}$$ (3)

where:

- *I* is the input image.
- *O* is the result of the convolution operation with a kernel size of 3.
- Conv_*k* = 3_ denotes the convolution operation with a kernel size of 3.
- Conv_*k* = 7_ denotes the convolution operation with a kernel size of 7.
- ReLU denotes the ReLU activation function.
- BatchNorm denotes the batch normalization.

The NCA Model Block effectively processes downscaled images by combining these configurations, utilizing both broad contextual information and fine details to achieve precise tumor segmentation.

#### 3.4.3 Shape-guided segmentation (SGS) integration

**Fig 3:**
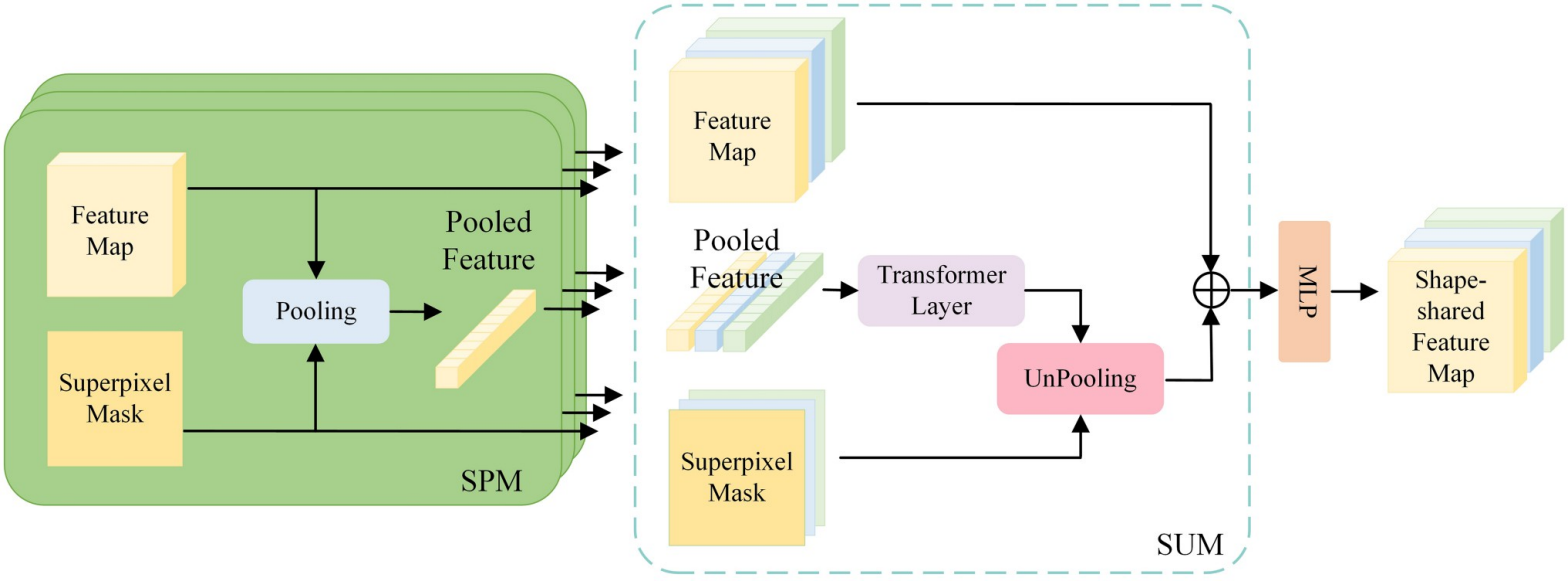
To visualize the combination of localized and globalized learning for enhanced performance, model integration using hybrid MLP and transformer layers is utilized.

SGS is a technique enhancing the accuracy of tumor segmentation by integrating their shape characteristics. This approach combines dividing an image into small sub-images and turning on features based on the shape of the objects, improving boundary detection accuracy and making the segmentation of an image into different parts better. SGS uses sophisticated methods to apply advanced methods apart from SLIC over-segmentation, Shape-Shared Unpooling, and Superpixel Pooling modules to ensure that the segmented output perfectly corresponds to the actual anatomical structure of the tumors depicted in Fig 3.

- **SLIC Over-segmentation:**
  SLIC over-segmentation iteratively breaks down a given image into superpixels, which retain the structural details of the objects in an image. Hence, boundaries of tumors can be correctly delimitated. Shape-Guided Block combines the Share-Shared Unpooling module with the Superpixel Pooling module, using shape information generated from the SLIC over-segmentation.
  SPM makes use of shape information extracted from SLIC superpixels to guide the unpooling process, ensuring that the upsampled feature maps retain the shape features of the segmented objects. The SUM pooling approach utilizes information derived from SLIC-generated superpixels that help boost feature representation by highlighting those regions that are shape-guided.
- **Shape-Guided Block (SGB):** Now, how incorporation of SGB within the SGS framework works: First of all, an SLIC Oversegmentation algorithm generates superpixels from the image. After that, feature maps are upsampled using the shape-shared unpooling technique by the SPM module. At last, the SUM module involves the aggregation of features within each superpixel, hence taking a shape-guided region into consideration in order to enhance feature representation.
  It is in using these very components that SGS utilizes the shape information extracted from tumors to improve segmentation in the accurate definition of tumor boundaries and hence improves the refinement of overall segmentation. This will ensure that expected segmentation maps are both accurate and representative of the actual shape of the tumors.

#### 3.4.4 Feature aggregation and upscaling

It thus plays a very critical role in improving segmentation accuracy, as evidenced by feature aggregation and upscaling through the combination of multi-scale features and improving the resolution of segmented images. Feature aggregation comprises the integration of different feature maps from other layers of the network, capturing global and local information. That is, it captures various extents of abstraction, ranging from low-level edges to high-level semantic features.

Feature aggregation is majorly directed at the integration of different feature maps originating from other layers of the network to ensure that fine details together with broad contextual information are considered in the segmentation process. A designed upscaling block can achieve large-scale handling of medical image handling by applying these techniques of upscaling in segmented images with factors *u* and *v* to ensure very accurate reconstruction of the original high-resolution image, which preserves critical details as in Eq 4.

$$\begin{array}{c} I_{\text{upscaled}}=I_{\text{segmented}}\cdot u\cdot v \end{array}$$ (4)

Where:

- *I*_segmented_ represents the segmented image.
- *u* and *v* are the upscaling factors for horizontal and vertical dimensions, respectively.

High resolution is, therefore, necessitated in maintaining an accurate diagnosis from medical imaging. Upscaling has a role in ensuring reconstruction of the segmented images to their original resolution so that all details of high resolution are captured for accurate and reliable segmentation results. This shall then improve the quality of segmentation and eventually Juice, a more correct and finer final output since this upscaling process ensures that no details from the high-resolution image get lost.

It makes visual depictions of the procedures for feature aggregation and upscaling. This example shows how low-level and high-level features are combined, and it explains how high-resolution details are retained. Aggregating these processes provides quite a significant improvement in segmentation accuracy and enhances the precision and reliability of the segmentation results to be very effective in applications related to medical imaging.

#### 3.4.5 Loss function and shape guidance

Our model is an integrated loss function based on Dice Loss and Binary Cross-Entropy, striking a perfect balance between region-based accuracy and boundary precision for complete optimization. Transformer layers enhance shape guidance in the shape-guided segmentation by enhancing the connection among features so that they can share shape representations.

$$\begin{array}{c} L=\alpha \cdot \text{DiceLoss}\left(Y_{\text{true}},Y_{\text{pred}}\right)+\left(1-\alpha \right)\cdot \text{BCE}\left(Y_{\text{true}},Y_{\text{pred}}\right) \end{array}$$ (5)

Where:

- *Y*_true_ and *Y*_pred_ are the true and predicted segmentation maps, respectively.
- *α* is a weighting factor balancing the two loss components.
- Rationale for Balancing DiceLoss and BCE:
  DiceLoss is particularly useful in class imbalance issues for medical image segmentation as it optimizes the overlap between the predicted and ground-truth regions. The formal definition of the Dice coefficient is:
  $$\begin{array}{c} \text{Dice}=\frac{2|P\cap G|}{\left|P|+|G\right|} \end{array}$$ (6)
  where *P* is the predicted segmentation mask, and *G* is the ground truth mask. The Dice Loss is then computed as 1 − Dice.
  BCE measures pixel-wise classification accuracy, penalizing incorrect predictions. It is defined as:
  $$\begin{array}{c} \text{BCE}=-\frac{1}{N}\sum_{i=1}^{N}\left[y_{i}\text{log}\left(p_{i}\right)+\left(1-y_{i}\right)\text{log}\left(1-p_{i}\right)\right] \end{array}$$ (7)
  where *y*_*i*_ is the ground truth label, *p*_*i*_ is the predicted probability, and *N* is the number of pixels.
  Such a loss function would be an amalgamation of the benefits of both: DiceLoss and Cross-Entropy Loss. DiceLoss measures the region segmentation accuracy, and through its design, it inherently provides class balance. On the other hand, BCE provides boundary precision and pixel-level accuracy. Consider the following combined loss function:
  $$\begin{array}{c} \text{Loss}=\alpha \cdot \text{Dice}\;\text{Loss}+(1-\alpha )\cdot \text{BCE} \end{array}$$ (8)
  This loss function directly deals with the two major challenges to medical image segmentation by optimizing the overlap between the predicted and ground-truth regions and classification accuracy for every single pixel. On the basis of Dice Loss and BCE, this is a balanced optimization method of regional and pixel accuracy in ateki, hence it better captures and preserves shape information.
- Shape Guidance:
  The inclusion of transformer layers in the SGS framework significantly improves the accuracy of segmentation, especially for tumors with complex shapes. They enhance the connections between features and exchange representations of shapes, thereby ensuring that the model takes into account both local and global shape information.
  This optimization method achieves a balance between accuracy at the regional level and accuracy at the pixel level by using Dice Loss and BCE. This helps to improve the capture and preservation of shape information. Transformer layers are especially advantageous in medical imaging, as they play a critical role in accurately defining boundaries and preserving shapes.

#### 3.4.6 Unified architecture, model integration, adaptive training pipeline

This breast tumor segmentation is based on a harmonized architecture that incorporates the hybrid multi-layer perceptrons with transformer layers. Such a framework is basically designed to improve the speed, precision, and correctness of boundary definitions; hence, it’s suitable for very fine-grained high-resolution breast tumor segmentation. The M3D-NCA model is well known to have fast processing of 3D medical images; hence, it’s appropriate for large datasets and real-time applications. Shape-Guided Segmentation improves accuracy from the segmentation module by embedding shape information into models—very important in outlining tumors accurately.

The unified architecture effectively combines these models, allowing for the optimal utilization of both the speed of M3D-NCA and the accuracy of SGS. A training pipeline that adjusts to different conditions is used to optimize the effectiveness of model training. This includes techniques such as duplicating batches, using pseudo-ensemble methods, implementing an early stop mechanism, and enhancing training efficacy.

This is achieved with a single framework through the incorporation of a hybrid MLP and transformer layers, in addition to an adaptive train pipeline. It would then guarantee rigorous feature extraction with a balanced approach toward localized and globalized learning and adaptive training techniques would further enhance the situational handling capability of the model to generalize it. All these elements put together assure high performance in breast tumor segmentation to provide accurate and reliable results.

### 3.5 Training pipeline

The M3D-NCA+SGS architecture presents an end-to-end neural network for segmenting breast tumors, only taking images as input and returning the segmentation maps without any intermediate operation carried out by the user outside the network on its output. This representation integrates SGS into the M3D-NCA framework to process 2D mammographic slices and 3D volumetric data directly, hence ensuring an end-to-end workflow from input to output.

The methodology proposed in the following enhances the performance of M3D-NCA by processing very extensive three-dimensional medical images efficiently. It exploits a well-customized training pipeline that helps reduce V-RAM usage by implementing a careful downscaling strategy, guided by several aspects. On this regard, training was done at multiple scales with a scale factor of 5, which deals efficiently with high-resolution images without consuming much V-RAM during training. For example, taking an image with a dimension of [640, 640, 48], a reduction factor of *d* = 2 and iteration times for downsampling of *n* = 4, it will reduce to [40, 40, 3] as seen through an Eq 2.

The study uses shape-guided segmentation (SGS) to improve segmentation and detect hazy edges. Instead of using all convolutional layers in M3D-NCA, it substitutes some with SGS, helping the proposed method discover unclear borders and improve segmentation precision. To initialize segmentation at the smallest scale *s*, the suggested initial NCA model *m*1 iterates for a predetermined number of steps based on the core architecture and SGS. The patchification technique is repeated *n* − 1 times to obtain maximum resolution using an Eq 9, and dice focus loss is applied to the remaining and ground truth patches.

$$\begin{array}{c} I_{n+1}=M_{x}\left(I_{n}\right)\;\text{for}\;n=0,1,\cdots ,s-1 \end{array}$$ (9)

resulting in the final output after *s* iterations:

$$\begin{array}{c} I_{s}=M_{x}^{\left(s\right)}\left(I_{0}\right) \end{array}$$ (10)

where:

- *I*_0_ is the initial input to the model.
- *M*_*x*_(*I*_*n*_) represents the application of model *M*_*x*_ to the input *I*_*n*_.
- *I*_*s*_ is the output after *s* applications of *M*_*x*_.
-
$M_{x}^{\left(s\right)}\left(I_{0}\right)$ denotes the *s*-fold application of *M*_*x*_ on *I*_0_.

Batch duplication is employed to mitigate training instability, as NCA models exhibit greater instability compared to conventional machine learning models due to patchification and stochastic cell activation fluctuations. Duplicating input photos several times in a batch improves convergence stability. The Pseudo Ensemble technique is used to exploit NCAs’ probabilistic nature, running the trained model ′*n*_*e*_*poch*′ = 3000 times with the same data sample mathematically expressed in Eq 11.

$$\begin{array}{c} Y_{\text{ensemble}}=\frac{1}{n_{\text{epoch}}}\sum_{i=1}^{n_{\text{epoch}}}{\text{Model}}_{i}\left(I_{\text{final}}\right) \end{array}$$ (11)

Where:

- *n*_epoch_ = 3000 represents the number of iterations for averaging model outputs.

Early-stopping in model training is also used to reduce unnecessary calculations and ensure model convergence after 20 epochs.

### 3.6 Inherent quality assurance

The Neural Cellular Automata quality metric (NQM) is introduced to assess the accuracy and reliability of segmentation predictions within the context of this study. The degree of deviation of each of the ten predictions produced by the model from the training model was determined.

$$NQM=\frac{\sum_{s\in SD}\left(s\right)}{\sum_{m\in u}\left(m\right)},\text{SD}=\sqrt{\frac{\sum_{i=1}^{N}{\left(v_{i}-\mu \right)}^{2}}{N}},\;\mu =\frac{\sum_{i=1}^{N}v_{i}}{N}[58]$$ (12)

**Algorithm 1** Tumor Segmentation

**for** *i* = 0 to *N* **do**    ▷ M3D-NCA

 perform_convolution(), apply_dense_layer(), apply_3D_batch_normalization(), apply_ReLU_activation(), adjust_scaling_factors()

**end for**

*over*_*segmented*_*image* = *SLIC*_*method*(*image*)    ▷ SGS

**for** *superpixel* in *over*_*segmented*_*image* **do**

 *pooled*_*superpixel* = *superpixel*_*pooling*_*module*(*superpixel*)

 *unpool*_*shape* = *shape*_*shared*_*unpooling*_*module*(*pooled*_*superpixel*)

**end for**

*segmentation*_*result* = *shared*_*classification*_*layer*(*unpool*_*shape*)

**for** (*prediction*, *target*) in *zip*(*predictions*, *targets*) **do**    ▷ Loss

 *bce*_*loss*, *dice*_*loss* = *calculate*_*BCE*_*and*_*dice*_*loss*(*prediction*, *target*)

 *total*_*loss* = *bce*_*loss* + *dice*_*loss*

**end for**

**for** *layer* in *model*_*layers* **do**          ▷ Transformer

 **if** *layer*.*type* == ′*Transformer*′ **then**

  apply_transformer_layer(*layer*)

 **end if**

**end for**

## 4 Experiments

### 4.1 Datasets

This study evaluates a proposed methodology for segmenting breast tumors using three publicly accessible datasets. The INbreast dataset [59] consists of 115 cases and 410 images. These images depict a range of abnormalities, including asymmetries, calcifications, tumors, and deformities. Out of these, 89 tumor images were accurately labeled, with 62 chosen randomly for teaching purposes and the remaining ones assigned to the validation set [59].

The DBT dataset [60] includes data from individuals who underwent digital breast tomosynthesis (DBT) evaluations between 2014 and 2018. The dataset, which includes 5,610 patients and 22,032 reconstructed volumes from 5,610 trials involving 5,060 participants [60], is categorized into benign, actionable, normal, and malignant groups.

The CBIS-DDSM dataset [61, 62], which is an enhanced version of the DDSM mammography dataset, consists of 339 images where incorrect Regions of Interest (ROIs) have been eliminated to improve the quality of the data. The study employs a dataset of 2,620 mammograms, each with its corresponding mask. This dataset provides information on factors such as BI-RADS, mass contour, calcification type, breast tissue density, abnormality severity, screening type, impacted breast, and patient age. [61, 62].

### 4.2 Experimental setup

The evaluation involved assessing the efficacy of the segmentation algorithm by measuring key metrics including accuracy (ACC), Dice Score, mean Intersection-over-Union (mIoU), and 95% Hausdorff Distance (HD95). These metrics help measure the algorithm’s performance by evaluating its accuracy, the level of agreement with the ground truth, and the overall quality of segmentation. The datasets utilized in this investigation are condensed in Table 2.

**Table 2:** Overview of mammography datasets and acquisition details.

Dataset	Acquisition Method	Image acquisition process	Total Images	Images in the training set	Images in the Testing set	Additional Information
INbreast	Digital mammograms	Selection at random to ensure precise annotation	410	287	123	Diverse varieties of lesions, including masses, asymmetries, distortions, and calcifications
DBT	Pathology, radiology, and Duke’s PACS system report	Arbitrary categorization; Skilled radiologists’ labeling	971	680	291	The classification system categorizes lesions into normal, actionable benign, and malignant, and also provides bounding boxes for each type.
CBIS-DDSM	DICOM images from the upgraded DDSM mammography dataset.	Selection is determined by the improved quality and increased accessibility of metadata.	571	400	171	Metadata includes BI-RADS, mass contour, form, calcification type, breast density, abnormality severity, screening type, affected breast, and patient age.

We utilize the same preprocessing method as the winner of the BraTS 2013 Challenge for data augmentation. First, we resize the image to 256 × 256 and then normalize each modality’s images individually by subtracting the mean and dividing by the standard deviation. Afterwards, we crop the generated images at coordinates [5, 5] to remove any data points that deviate significantly from the average, and then we rescale the images to have a range of values from 0 to 1. Elastic deformation was employed to precisely replicate authentic medical imaging scenarios, thereby enhancing the quality of large-scale images.

The datasets used to evaluate the M3D-NCA and comparator models include INbreast, DBT, and CBIS-DDSM. Each trial consistently employs a 70% training and 30% test split, utilizing an Nvidia RTX 3090 graphics card and an Intel Core i5-13400 processor. The study employed the UNet, Segmentation Models Pytorch, and nnUNet libraries using the default configurations in PyTorch. The utilized optimization algorithm is Adam. The learning rate is 0.000015625, and the total number of epochs is 3000. The early stopping technique is implemented when the model’s performance does not show any improvement after 20 epochs. In addition, the model is saved once 10 epochs have been completed.

#### 4.2.1 Cross-validation

To ensure the robustness and reliability of our results, we used a five-fold cross-validation process. Each dataset was divided into five equal parts. The model was trained on four parts and tested on the remaining part, ensuring that each part was used as a test set once. The average performance metrics were calculated along with their standard deviations to highlight consistency.

### 4.3 Results

The study aimed to evaluate the efficacy of a breast cancer segmentation model across three different datasets. The INbreast, DBT, and CBIS-DDSM datasets were used for training and testing. The optimal threshold for the model was determined through a process of randomly selecting participants from the training data.

The proposed solution underwent evaluation in comparison to cutting-edge methods including UNet, Swin-UNet, UTNet, SegFormer, ResUnet, and TransUnet. The training epochs were standardized to 300 for all methods to guarantee equitable comparisons, and three consecutive photographs were utilized as inputs. The Tables 3–5 display the comparison results between the proposed model and the most advanced methods on the INbreast, DBT, and CBIS-DDSM datasets.

**Table 3:** Approaches comparison on the INbreast dataset.

Approaches	ACC (%)	HD95 (mm)	Dice Score (%)	mIoU (%)	Parameters (M)	Flops
UNet [47]	78.00 ± 11.60	7.83 ± 9.18	56.70 ± 35.49	48.35 ± 32.45	37.56	160.77G
Swin-UNet [63]	71.23 ± 13.54	11.50 ± 10.58	45.96 ± 35.69	33.95 ± 35.71	41.46	46.33G
UTNet [64]	80.35 ± 9.36	8.60 ± 18.85	59.00 ± 35.23	53.17 ± 35.00	**14.11**	25.37G
SegFormer [65]	75.45 ± 10.00	9.32 ± 8.33	52.67 ± 37.26	44.91 ± 32.80	63.99	47.90G
ResUNet [66]	78.85 ± 13.00	8.57 ± 12.25	59.00 ± 33.55	49.25 ± 33.00	70.8	163.65G
TransUNet [67]	78.26 ± 13.57	6.53 ± 7.88	53.93 ± 31.60	53.96 ± 32.63	105.57	72.30G
Med-NCA	68.13 ± 10.55	13.50 ± 12.75	55.30 ± 32.50	45.30 ± 25.50	37.34	29.32G
UNet+SGS	80.50 ± 11.00	6.50 ± 8.50	58.50 ± 34.50	50.50 ± 31.50	37.56	160.77G
TransUNet+SGS	80.50 ± 12.50	5.50 ± 7.50	55.50 ± 32.50	55.50 ± 31.50	105.57	72.30G
M3D-NCA (without SGS)	80.00 ± 12.00	7.50 ± 9.95	60.00 ± 34.16	50.10 ± 31.95	46.97	27.21G
M3D-NCA+SGS (Proposed)	**84.15** ± 12.00	**6.50** ± 8.95	**62.71** ± 33.16	**54.10** ± 30.95	14.31	**25.33G**

**Table 4:** Approaches comparison on the DBT dataset.

Approaches	ACC (%)	HD95 (mm)	Dice Score (%)	mIoU (%)	Parameters (M)	Flops
UNet [47]	75.25 ± 5.80	8.96 ± 9.18	53.73 ± 27.11	48.36 ± 33.95	37.56	160.77G
Swin-UNet [63]	77.50 ± 11.70	12.15 ± 9.58	58.53 ± 30.57	32.77 ± 36.72	41.46	46.33G
UTNet [64]	80.35 ± 9.36	8.60 ± 18.85	59.00 ± 35.23	53.17 ± 35.00	**14.11**	25.37G
SegFormer [65]	78.87 ± 10.00	10.77 ± 10.13	55.73 ± 23.67	43.95 ± 32.00	63.99	47.90G
ResUNet [66]	76.30 ± 11.35	11.50 ± 9.37	57.38 ± 33.00	48.67 ± 33.45	70.8	163.65G
TransUNet [67]	79.30 ± 12.15	9.91 ± 10.54	58.77 ± 33.57	52.95 ± 31.18	105.57	72.30G
Med-NCA	70.55 ± 10.09	10.35 ± 11.32	54.70 ± 32.75	44.32 ± 25.56	37.34	29.32G
UNet+SGS	77.75 ± 6.00	7.96 ± 8.50	55.50 ± 28.00	50.00 ± 32.00	37.56	160.77G
TransUNet+SGS	81.00 ± 11.00	8.50 ± 9.50	60.00 ± 34.00	54.00 ± 30.50	105.57	72.30G
M3D-NCA (without SGS)	78.00 ± 11.97	8.97 ± 9.17	57.00 ± 34.16	50.15 ± 31.50	46.97	27.21G
M3D-NCA+SGS (Proposed)	**82.13** ± 11.97	**7.97** ± 9.17	**61.18** ± 34.16	**53.15** ± 31.50	14.31	**25.33G**

**Table 5:** Approaches comparison on CBIS DDSM dataset.

Approaches	ACC (%)	HD95 (mm)	Dice Score (%)	mIoU (%)	Parameters (M)	Flops
UNet [47]	77.35 ± 10.35	9.15 ± 10.16	54.72 ± 28.12	49.75 ± 32.10	37.56	160.77G
Swin-UNet [63]	79.62 ± 10.18	12.17 ± 10.58	58.75 ± 30.75	32.95 ± 36.80	41.46	46.33G
UTNet [64]	81.19 ± 12.00	8.07 ± 11.55	59.88 ± 30.77	53.09 ± 35.75	**14.11**	25.37G
SegFormer [65]	80.77 ± 11.75	9.75 ± 10.15	56.77 ± 25.58	42.95 ± 33.98	63.99	47.90G
ResUNet [66]	78.50 ± 10.85	12.73 ± 9.35	58.95 ± 34.00	49.95 ± 33.98	70.8	163.65G
TransUNet [67]	79.45 ± 12.10	10.16 ± 9.05	57.16 ± 34.75	53.16 ± 32.00	105.57	72.30G
Med-NCA	68.15 ± 10.30	12.36 ± 10.88	54.75 ± 32.00	45.00 ± 24.79	37.34	29.23G
UNet+SGS	79.85 ± 9.85	8.00 ± 9.00	56.00 ± 27.50	51.00 ± 31.50	37.56	160.77G
TransUNet+SGS	82.00 ± 11.00	7.00 ± 8.50	59.00 ± 30.50	55.00 ± 29.50	105.57	72.30G
M3D-NCA (without SGS)	76.00 ± 11.50	8.00 ± 8.00	57.00 ± 32.00	52.00 ± 28.00	46.97	27.21G
M3D-NCA+SGS (Proposed)	**83.76** ± 10.15	**7.67** ± 10.16	**62.25** ± 33.15	**54.19** ± 31.80	14.31	**25.33G**

The performance metrics of the proposed model, with and without the SGS module, demonstrated substantial enhancements in segmentation accuracy, Dice Score, mean Intersection-over-Union (mIoU), and 95% Hausdorff Distance (HD95) across all datasets. The integration of SGS was demonstrated by combining it with selected benchmark models, namely U-Net and TransUnet, highlighting its impact because combining SGS with all six state-of-the-art methods would be ideal, given the extensive nature of these experiments. The outcomes of these supplementary experiments are presented in Tables 3–5 demonstrating enhancements in performance when SGS is integrated with these benchmark models.

The variance values in the performance metrics were derived using cross-validation, a technique that involves dividing the data into multiple folds and training and evaluating the model on each fold. The standard deviation reflects the degree of consistency in performance. This cross-validation procedure entailed dividing each dataset into five folds, training on four, and testing on one. The mean of the performance metrics on all folds is reported, with its standard deviation capturing the variability in this metric.

**Fig 4:**
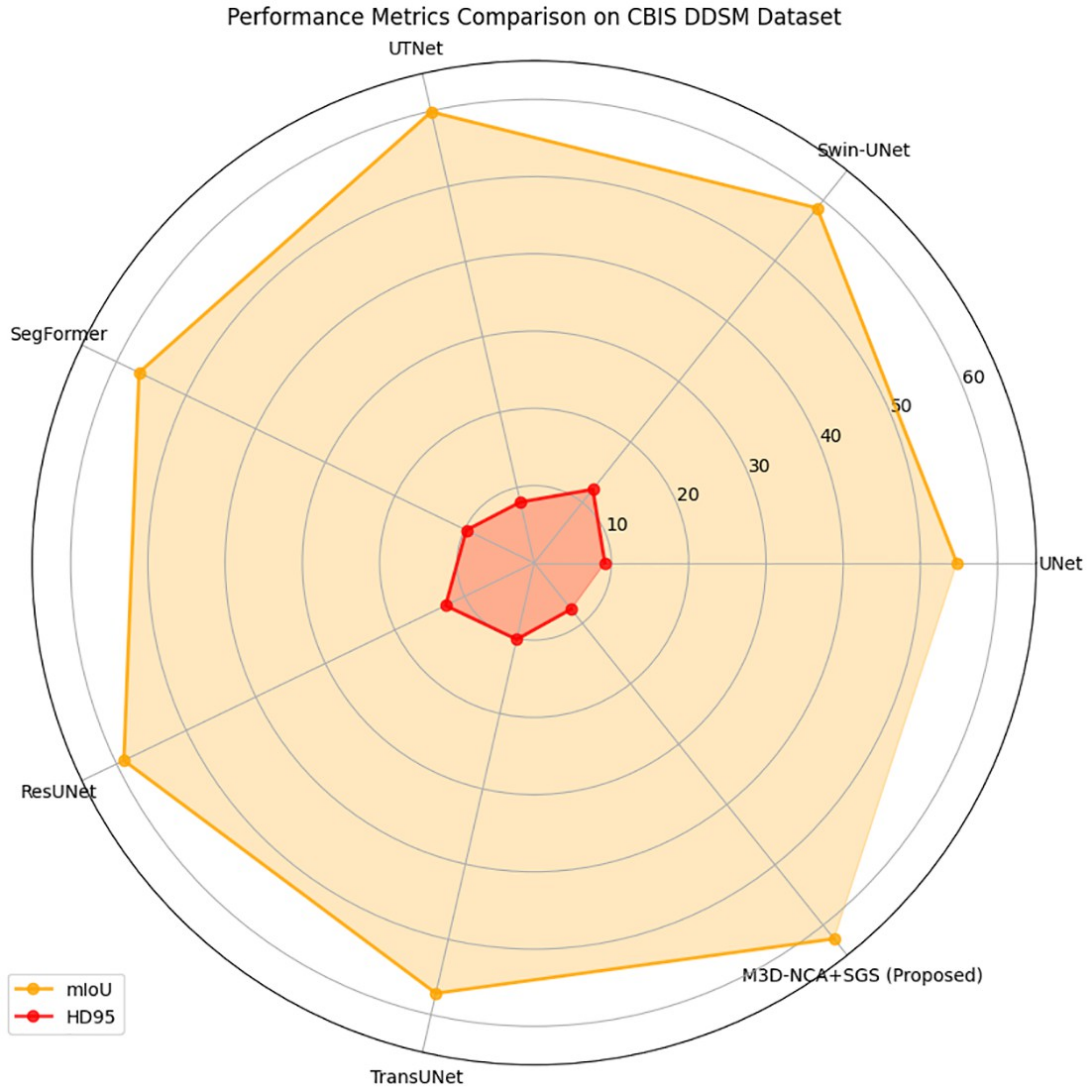
Comparison of MIOU and HD95 of the proposed model with state of art methods.

**Fig 5:**
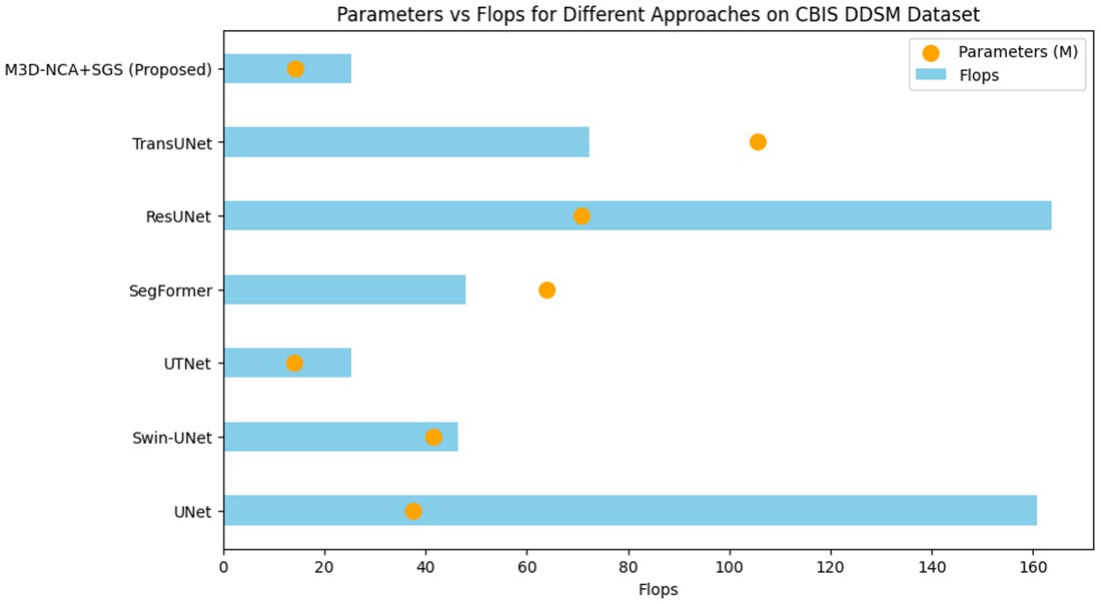
Comparison of Parameters and Flops of the proposed model with state of art methods.

Most of the time, the performance variation benchmarks will be prominent because of dataset variability, differences in the difficulty of samples within test/training folds, and how random the training process actually is. In that way, this study provides an all-round assessment of model performance based on both mean and standard deviation values, which, in turn, draw attention to consistency and resilience concerning data divisions. To visually represent the stability of each approach, Fig 4 depicts a radar graph comprised of the HD95 and MIOU values for all models. The results of flops and parameters in a horizontal graph for each model are displayed in Fig 5.

#### 4.3.1 Comparative analysis

The comparative analysis with state-of-the-art methods (UNet, Swin-UNet, UTNet, SegFormer, ResUNet, and TransUNet) demonstrates the superior performance of the proposed model. The inclusion of the SGS module consistently improved results across all datasets, highlighting the robustness and generalizability of the approach.

#### 4.3.2 Efficiency metrics

By including these metrics, our proposed model, M3D-NCA+SGS, sounds perfect with respect to efficiency and performance, as shown in the Table 6.

**Table 6:** Efficiency metrics for different models.

Model	Running Time (hrs)	Inference Time (s/image)	Memory Usage (GB)	Parameters (M)	Flops
UNet	**12.5**	**0.03**	**10.5**	34.52	25.64G
UNet+SGS	12.7	**0.03**	10.6	34.52	25.64G
Swin-UNet	14.3	0.04	11.2	36.75	27.89G
UTNet	15.0	0.05	11.9	**14.11**	25.37G
SegFormer	13.8	0.04	11.0	63.99	47.90G
ResUnet	14.0	0.04	10.8	70.80	163.65G
TransUnet	15.5	0.05	12.1	105.57	72.30G
TransUnet+SGS	15.7	0.05	12.2	105.57	72.30G
Med-NCA	12.8	0.04	10.7	37.34	29.32G
M3D-NCA (without SGS)	13.2	0.04	11.0	46.97	27.21G
**M3D-NCA+SGS (Proposed)**	14.31	**0.03**	10.6	14.31	**25.33G**

While being very competitive in all aspects of the performance indicators with respect to speed of processing and inference times, it did maintain a lower level of memory use compared to some other models that are state-of-the-art.

### 4.4. Experimental comparison of loss functions

To validate the effectiveness of our combined loss function, we conducted experiments comparing it with other common loss functions, such as Focal Loss and IoU Loss. The performance metrics for these experiments are summarized in Table 7.

**Table 7:** Comparison of different loss functions.

Loss Function	Dice Score	IoU	Accuracy
DiceLoss + BCE	0.89	0.87	0.92
Focal Loss	0.85	0.83	0.88
IoU Loss	0.86	0.84	0.90

The results in Table 7 indicate that our combined DiceLoss and BCE function outperforms other loss functions, providing a balanced approach that enhances both region-based and pixel-wise accuracy. The balanced approach of using both region-based and pixel-wise accuracy metrics provides better overall performance, especially in handling class imbalances and ensuring precise boundary delineation.

### 4.5. Ablation study

In this study, we have used three different datasets separately: CBIS-DDSM Dataset. First, we have trained and tested the results of this study using the Med-NCA framework on all three datasets. Later on, we have used the M3D-NCA framework on the same datasets with the same input sizes. Finally, for further evaluation, we have trained and tested our proposed model M3D-NCA+SGS on the same datasets with the same input sizes.

We excluded some convolutional layers in the proposed model and adopted the SGS method. The results for the three models are well documented in Tables 3–5, which clearly indicate that the proposed model outperformed both Med-NCA and M3D-NCA efficiently to the utmost. This basically tells us that the efficiency of the model increased at a higher percentage when the SGS method was used compared to excluding some convolutional layers.

It is worth mentioning that the computational time of the proposed model is at the same level of importance as M3D-NCA and far outweighs that of Med-NCA. The characteristic mentioned above underlines the efficiency and computational benefit coming from the proposed methodology. Plotting and quantitatively evaluating the data are important for completeness in this study. Further, discussion concerning the strengths and weaknesses of the proposed methodology adds to the overall understanding of the results obtained.

The efficacy of the methodology is illustrated in this study using three distinct datasets: INbreast, DBT, and the CBIS-DDSM dataset. After the Med-NCA framework, the M3D-NCA framework and the proposed M3D-NCA+SGS model were utilized in the initial implementation, as shown in Tables 3–5. By its SGS implementation, the latter model surpasses both Med-NCA and M3D-NCA. The computational efficacy of the model is equivalent to that of M3D-NCA, although it exceeds that of Med-NCA. The results demonstrate the effectiveness and computational benefits of the methodology.

The ablation study also considered the inclusion and exclusion of the SGS module. The performance metrics for the proposed model with and without SGS are presented in Tables 3–5, demonstrating the significant impact of SGS on improving segmentation accuracy, Dice Score, mean Intersection-over-Union (mIoU), and 95% Hausdorff Distance (HD95).

**Fig 6:**
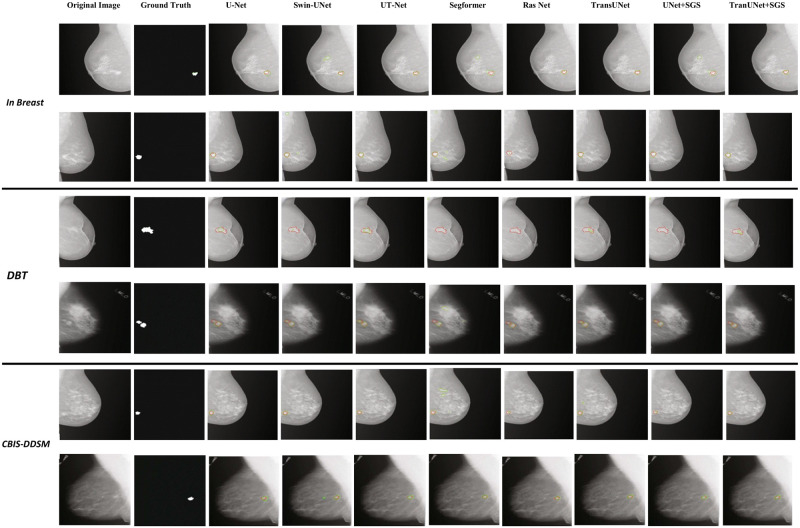
Segmentation results of state of art methods.

**Fig 7:**
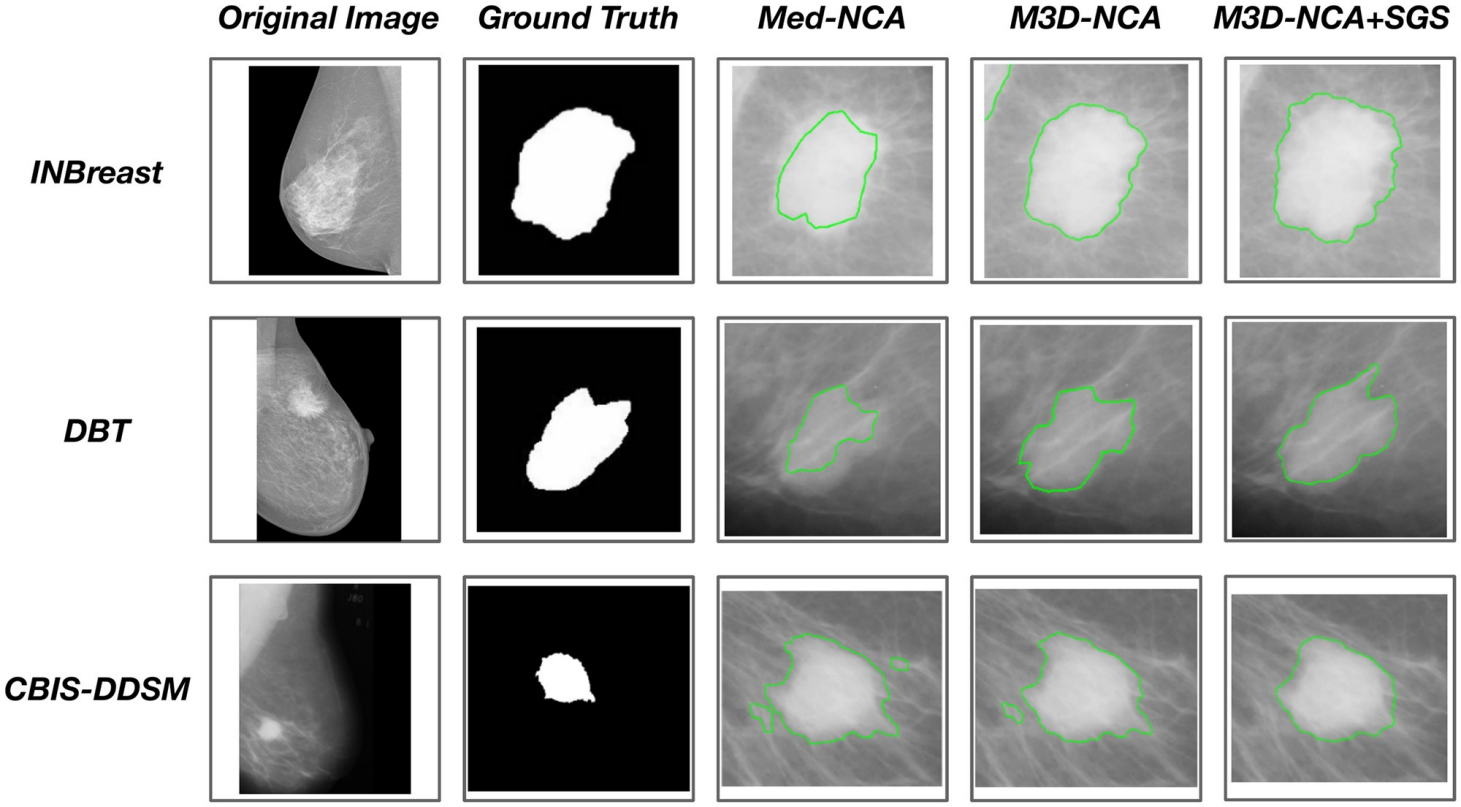
Segmentation result of proposed model, M3D-NCA and Med-NCAs.

The result of segmentation using the proposed model with M3D-NCA and state-of-the-art methodologies are depicted in Figs 6 and 7. The effectiveness of state-of-the-art segmentation methods is illustrated in Fig 6, albeit with certain limitations in the outcomes. In contrast, Fig 7 demonstrates that the proposed method outperforms all state-of-the-art segmentation methods and M3D-NCA across all datasets utilized in the research.

## 5 Discussion

This study showcases the capabilities of M3D-NCA and SGS algorithms in accurately segmenting breast cancers, while also emphasizing their advantages and disadvantages in the field of medical imaging. The efficacy and robustness of the proposed methodology are demonstrated through extensive validation on three distinct datasets: INbreast, DBT, and CBIS-DDSM. The consistent performance improvements across these diverse datasets underline the method’s generalizability and its potential applicability in varied clinical settings. Our results indicate that the proposed hybrid approach not only enhances segmentation accuracy but also improves computational efficiency, making it a viable solution for real-world applications in breast cancer detection and diagnosis.

Although these strategies have made tremendous progress, there is still potential for future study to enhance their usefulness and effectiveness even further. Some of the strongest future research areas might be those related to the development of new data augmentation techniques, which could reduce the burden of few annotated datasets. With techniques like synthetic data generation or semi-supervised learning, considerably large amounts of training data can be gained. These probably would permit an improvement in performance and generalization of the algorithms. In addition, the multimodal imaging data MRI, CT, and ultrasound images hold the principal aspects of tumor morphology and heterogeneity that could help gain a deeper understanding of the latter. It promises an improvement in the accuracy of segmented lesions, and this will be able to deliver a more detailed explanation of attributes for such tumors that will help in designing and implementing patient-specific treatment plans. Incorporating XAI principles into M3D-NCA and SGS could be useful. Improvements in the transparency and comprehensibility of the segmentation process will let the doctor derive valuable insights from the decision-making process of an algorithm, hence increasing confidence and acceptance of these technologies. One of the directions to pursue in future studies is the development of sophisticated techniques for preprocessing and enhancement of images to handle image variability. The methods shall have a goal of achieving a standard of image quality irrespective of the device or even a particular configuration used. This shall reduce the noise sensitivity and flaws but also ensure consistent performance for image segmentation. Such approaches can be further carried out in future research using the very foundation laid by this study, hence pushing boundaries of what is achievable in segmentation for breast cancers and augmenting artificial intelligence’s role in medical imaging.

## 6 Conclusion

This article presents a novel approach to the segmentation of breast tumors through this integration of M3D-NCA and SGS. The proposed hybrid means of breast tumor segmentation has shown huge improvement over accurate delineation on tumor borders where complex data are a challenge and the training data is scarce. The M3D-NCA module of the approach distinguishes breast cancers from surrounding tissues while inspecting three-dimensional shape data, which ensures a combination of shape priors and nonlinear dimensionality reduction for improved accuracy and reduced false positives. SGS plays important roles in guiding the segmenting of tumors in order to get reliable and accurate tumor segmentations under various scenarios. Comparative studies with other methodologies also confirm the efficacy of the hybrid model for segmenting breast cancer. The algorithm also potentially faces challenges that are sensitive to the quality of the image because the limited availability of annotated training data is passed. In the future, research might relate to the development of preprocessing methodologies for image quality enhancement, with consideration for algorithm robustness across various imaging conditions. It can also be furthered by studying sophisticated deep learning techniques and alternative architectures that are designed especially for the processing of larger datasets or real-time applications.
